# Comparative assessment of five trials of universal HIV testing and treatment in sub‐Saharan Africa

**DOI:** 10.1002/jia2.25048

**Published:** 2018-01-08

**Authors:** Delphine Perriat, Laura Balzer, Richard Hayes, Shahin Lockman, Fiona Walsh, Helen Ayles, Sian Floyd, Diane Havlir, Moses Kamya, Refeletswe Lebelonyane, Lisa A Mills, Velephi Okello, Maya Petersen, Deenan Pillay, Kalpana Sabapathy, Kathleen Wirth, Joanna Orne‐Gliemann, François Dabis, the Universal Test and Treat Trials Consortium (UTC)

**Affiliations:** ^1^ Inserm, Bordeaux Population Health Research Center, UMR 1219 University Bordeaux Bordeaux France; ^2^ Inserm, ISPED Bordeaux Population Health Research Center, UMR 1219 Bordeaux France; ^3^ Africa Health Research Institute Somkhele KwaZulu‐Natal South Africa (ANRS TasP trial); ^4^ University of California San Francisco San Francisco CA USA (SEARCH trial); ^5^ University of Massachusetts Amherst Amherst MA USA; ^6^ Department of Infectious Disease Epidemiology London School of Hygiene & Tropical Medicine London United Kingdom (PopART trial); ^7^ Harvard School of Public Health Boston MA USA (BCPP trial); ^8^ Botswana Harvard AIDS Institute Partnership Gaborone Botswana (BCPP trial); ^9^ Brigham and Women's Hospital Boston MA USA (BCPP trial); ^10^ Clinton Health Access Initiative Boston MA USA (MaxART trial); ^11^ Department of Clinical Research London School of Hygiene & Tropical Medicine London United Kingdom (PopART trial); ^12^ Zambart Lusaka Zambia; ^13^ Makerere University School of Medicine Uganda (SEARCH trial); ^14^ Ministry of Health, Republic of Botswana Gaborone Botswana (BCPP); ^15^ Centers for Disease Control Gaborone Botswana (BCPP trial); ^16^ Ministry of Health, Kingdom of Swaziland Mbabane Swaziland (MaxART trial); ^17^ University of California Berkeley School of Public Health Berkeley CA USA (SEARCH trial); ^18^ Department of Infection University College London London United Kingdom (ANRS TasP trial); ^19^ Department of Infection University College London London United Kingdom (ANRS TasP trial)

**Keywords:** HIV, universal test and treat, randomized trials, comparative assessment, protocols, sub‐Saharan Africa

## Abstract

**Design:**

Universal voluntary HIV counselling and testing followed by prompt initiation of antiretroviral therapy (ART) for all those diagnosed HIV‐infected (universal test and treat, UTT) is now a global health standard. However, its population‐level impact, feasibility and cost remain unknown. Five community‐based trials have been implemented in sub‐Saharan Africa to measure the effects of various UTT strategies at population level: BCPP/YaTsie in Botswana, *Max* ART in Swaziland, HPTN 071 (PopART) in South Africa and Zambia, SEARCH in Uganda and Kenya and ANRS 12249 TasP in South Africa. This report describes and contrasts the contexts, research methodologies, intervention packages, themes explored, evolution of study designs and interventions related to each of these five UTT trials.

**Methods:**

We conducted a comparative assessment of the five trials using data extracted from study protocols and collected during baseline studies, with additional input from study investigators. We organized differences and commonalities across the trials in five categories: trial contexts, research designs, intervention packages, trial themes and adaptations.

**Results:**

All performed in the context of generalized HIV epidemics, the trials highly differ in their social, demographic, economic, political and health systems settings. They share the common aim of assessing the impact of UTT on the HIV epidemic but differ in methodological aspects such as study design and eligibility criteria for trial populations. In addition to universal ART initiation, the trials deliver a wide range of biomedical, behavioural and structural interventions as part of their UTT strategies. The five studies explore common issues, including the uptake rates of the trial services and individual health outcomes. All trials have adapted since their initiation to the evolving political, economic and public health contexts, including adopting the successive national recommendations for ART initiation.

**Conclusions:**

We found substantial commonalities but also differences between the five UTT trials in their design, conduct and multidisciplinary outputs. As empirical literature on how UTT may improve efficiency and quality of HIV care at population level is still scarce, this article provides a foundation for more collaborative research on UTT and supports evidence‐based decision making for HIV care in country and internationally.

## 1 Introduction

In recent years, the debate regarding “treatment versus prevention” for HIV infection has shifted with adoption of the concept of “treatment‐as‐prevention” (TasP). Together with other compelling evidence from observational, ecological and modelling studies 1, 2, 3, 4, 5, the landmark HIV Prevention Trial Network (HPTN) 052 trial showcased TasP as a promising means to curb the global HIV epidemic. Indeed, the use of antiretroviral therapy (ART) was shown to be associated with a 93% reduction in HIV sexual transmission in HIV serodiscordant stable couples in whom the HIV‐positive partner was randomized to immediate initiation of ART irrespective of his/her individual clinical needs 6. Since then, the scientific rationale for TasP has expanded. In 2015, two randomized clinical trials showed that initiating ART as soon as possible after HIV diagnosis (regardless of disease stage) also yielded strong clinical benefits to the individual 7, 8.

The concept of TasP has been replaced by that of universal HIV testing and treatment (UTT), which offers HIV counselling and testing to an entire population and ART to all those HIV‐infected 9. In 2015, the World Health Organization (WHO) recommended that ART should be offered to all individuals diagnosed with HIV regardless of their CD4 count and clinical staging 10, and the Joint United Nations programme on HIV/AIDS (UNAIDS) proposed their 90‐90‐90 targets for HIV treatment scale‐up by 2020 (namely, that 90% of HIV‐infected persons know their status; that 90% of those who know their positive HIV status continue on an efficacious ART regimen; and that 90% of those on ART have undetectable HIV‐1 RNA) 11. Thus, the remaining research questions are “What are the feasibility, effects and costs of UTT on a population level, and how do we best implement this strategy?” Maximizing the coverage of HIV testing, effective linkage and retention in care, rapid access to and initiation of ART, and strong adherence for viral suppression are required to achieve the full potential impact of UTT 12, 13, 14, both on individual health outcomes for people living with HIV and on HIV incidence reduction.

Mathematical models have sought to predict the long‐term population‐level impact of UTT strategies on the epidemic 15, 16. All models have indicated that UTT could reduce HIV‐transmission, but the estimated impact of the UTT strategy on the reduction of HIV incidence ranged from 35% to 54% in the short‐term and from 32% to 74% in the longer term 16. Furthermore, these models vary greatly in structure and in parametric assumptions and rely on context‐specific variables, such as eligibility criteria for ART initiation, uptake and coverage of HIV testing, linkage to care, treatment initiation and adherence 13. Modelling the potential impact of UTT offered necessary but insufficient data points for national policy makers trying to determine how to successfully take UTT to scale in their unique contexts. As noted by the HIV Modelling Consortium, modelling is used to investigate the *potentia*l impact of UTT; empirical evidence from large randomized trials is needed to further assess the feasibility of rolling out UTT to whole communities 17.

As such, in a context where HIV funding is flat, obtaining evidence‐based data on UTT long‐term impact, cost‐effectiveness, feasibility, sustainability and acceptability at local and national levels is critical 15, 18. To fulfil this need, five community‐based trials were designed and implemented in the past five years in Southern and Eastern Africa. They were initiated before the WHO issued its recommendation for universal treatment 19, i.e. in a period of more restricted ART use 20. The common aim of these trials was to evaluate the effectiveness of UTT on HIV control in some of the most affected populations in sub‐Saharan Africa 21, 22, 23, 24, 25. In addition to measuring effects of UTT on HIV incidence, the studies are expected to provide critical data on the feasibility and cost of implementation of UTT (as well as more vs. less successful approaches to implementation), and thus to guide future policy and practice, by providing evidence for optimal scale‐up approaches 12.

Although the data collection of four out of five trials is not completed yet, the ongoing UTT trials have accumulated practical experience and data which could prove vital to large‐scale rollout of UTT strategies. The investigators of the five ongoing UTT randomized population‐based studies have agreed to collaborate as the Universal Test and Treat Trials Consortium (UT^3^C). This group shares protocols, field experiences and early data, to generate consensus statements when appropriate, and ultimately to facilitate joint analyses that can inform public policy and resource allocation.

The aim of this first paper is to describe, compare and contrast the contexts, research designs, intervention packages, themes explored and adaptations, that characterize these five ongoing large‐scale studies on UTT in Southern and Eastern Africa. This comparison will facilitate a better understanding of the results of these studies, when they are available, and may assist in understanding any differences found.

## 2 Methods

### 2.1 Design

We conducted a comparative assessment of the five large‐scale UTT randomized studies conducted in sub‐Saharan Africa: the ANRS 12,249 TasP (ANRS TasP) trial in South Africa 21, the *Max*ART study in Swaziland 22, the HPTN 071 (PopART) trial in South Africa and Zambia 23, the SEARCH trial in Uganda and Kenya 24 and the BCPP/YaTsie (BCPP) trial in Botswana 25. UTT trials gathered as a consortium (UT^3^C) constituted of two to three representatives per trial, including a trial principal investigator. After sharing trial protocols, we screened for essential study characteristics and consensually decided which should be compared and at what level of detail. Then we highlighted and interpreted differences and commonalities across the trials. Over a year and a half period, information was exchanged via emails (twice monthly), teleconferences (once monthly) and a half‐day meeting held prior to the manuscript submission. The first and last authors of the present manuscript led this collaborative work.

### 2.2 Sources of information

We first extracted information from the trial protocols: BCPP V4.0 (7 December 2015 for Evaluation Protocol, and 15 April 2016 for Intervention Protocol) 26, *Max*ART V2.0 (7 September 2015) 22, PopART V3.0 (16 November 2015) 23, SEARCH V7.0 (5 February 2016) 27, ANRS TasP V3.0 (12 March 2015) 21. We also extracted data from amendments to the trial protocols and sought input (including confirmation of accuracy) from study investigators where necessary. Finally, we included data from the study baseline and from national surveys (e.g. national HIV prevalence surveys, national Demographic and Health surveys, national Population and Housing censuses, national Poverty surveys, national Labour surveys, national UNAIDS global AIDS response reports, statistics of the United Nations Educational, Scientific and Cultural Organization, WHO country statistical profiles, World Bank reports).

### 2.3 Analysis

We identified study characteristics that were deemed essential for comparing the UTT trials and organized them in five categories: (1) Trial contexts: key demographic and socio‐economic characteristics of the populations living in the trial areas, health services available in the trial areas, and their HIV epidemiological profiles; (2) Trial research methodologies: trial design, primary outcome, populations and other methodological considerations; (3) Trial intervention packages: services delivered in the trial intervention and control arms as well as national standard of care; (4) Trial themes: areas of interest of the trials and data collected for secondary outcomes; (5) Trial adaptations: modifications of the trial designs and interventions during the conduct of the trials, especially in response to evolving treatment guidelines. The identification of those five categories as well as the interpretation of the results was done collectively, until a consensus was reached within the cross‐study collaboration.

## 3 Results

### 3.1 Trial contexts

Table 1 characterizes the trial population and their contexts at baseline (See Table S1 for national indicators). The trials vary in terms of the demographic composition. For example in SEARCH, half of the population living in the trial area is less than 15 years old (unpublished trial data), whereas in the South African study sites of PopART,26% of people are aged ≤15 years old (unpublished trial data). The trial contexts also differ in the economies involved (e.g. unemployment, poverty) as well as the education level of their populations. The proportion of adults who had no secondary school education ranged from 13% in the South African communities of PopART (unpublished data) to 71% in the SEARCH communities 28. While all trials are conducted in areas which suffer from generalized HIV epidemics, the baseline prevalence of HIV also varied and was estimated to be 30% TasP trial sites 29, 29% in BCPP 30, 29% in *Max*ART 31, 22% in the South African sites of the PopART (unpublished data), 21% in the Zambian sites of PopART (unpublished data), 20% in SEARCH Kenyan communities 32, and is less than 10% in the SEARCH Ugandan communities 32. Finally, important disparities are observed across trials in relation to the extent of development of the HIV health systems at baseline. The *Max*ART and BCPP trials are being implemented in areas characterized by a relatively high ART coverage with, respectively, 85% 33 and 73% 30 of the adult population over 15 years old on ART. In SEARCH, an estimated 57% of people ≥15 years old are on ART at baseline 32. TasP and PopART trials accounted for the smallest ART coverages with less than half of the population on ART in their study sites (38% among >15 years old in TasP 34, 30% and 39% among 18 to 44 years old in the South African and Zambian PopART communities (unpublished work)). Despite significant progress of the local governments in fighting the epidemics through a series of strategic and operational plans, the overall health sector response and capacity have not yet met the needs of the local population in all these settings. The epidemics continue to spread along socio‐economic development fault lines such as poverty, gender inequality, unemployment and lack of adequate social protection among others 35.

**Table 1:** Trial background characteristics at baseline

Trial indicators	BCPP	*Max*ART	PopART	SEARCH	ANRS TasP
Census and demographic					
Residency	Rural and peri‐urban	Peri‐urban and rural	Peri‐urban	Rural	Rural
Young in population living in trial area (%)^b^	42% aged <16 or >64 years old^b^ ^36^	38% aged <15 years old^c^ (2013) 37	SA: 26% aged ≤15 years old^b^ Z: 40% aged ≤15 years old^b^	50% aged <15 years old^b^	30% aged <15 years old^c^ (2013) 38
Median age of research study population (years)^a^	40 years old [IQR: 33 to 48]^a^	33 years old [IQR: 24 to 45]^a^	27 years old [IQR: 22 to 33]^a^	29 years old [IQR: 20 to 43]^a^ ^28^	32 years old [IQR: 22 to 52]^a^ ^34^
Female in research study population (%)^a^	64%^a^	64%^a^	SA: 69%^a^ Z: 72%^a^	53%^a^ ^28^	70%^a^ ^29^
Labour and poverty					
Unemployed working‐age population (%)	59%^a^	42%^c^ (2013) 39	SA: 72%^a^ Z: 77%^a^	5%^a^ ^28^	90%^a^ ^34^
Population living under the national poverty lines (%)	15%^c^ (2010) 40	63%^c^ (2009) 41	SA: 54%^c^ (2010) 42 Z: 46%^c^ (2010) 43	U: 19%^c^ (2012) 44 K: 46%^c^ (2005) 45	54% (2010)^c^ ^42^
Education and health					
Adult population with primary education or less (%)	31%^a^	31%^a^	SA: 13%^a^ Z: 35%^a^	71%^a^ ^28^	41%^a^ ^34^
HIV prevalence (%)	29% among 16 to 64 years old^a^ (2013) 30	29% among >15 years old^c^ (2015) 31	SA: 22% among 18 to 44 years old^a^ (2014) Z: 21% among 18 to 44 years old^a^ (2014)	SW‐U: 7% among ≥15 years old^a^ (2013) 32 E‐U: 3% among ≥15 years old^a^ (2013) 32 K: 20% among ≥15 years old^a^ (2013) 32	30% among >15 years old^a^ (2012) 29
HIV+ population on ART (%)	73% among >15 years old^a^ ^30^	85% among >15 years old^c^ (2013) 33	SA: 30% among 18 to 44 years old^a^ Z: 39% among 18 to 44 years old^a^	57% among ≥15 years old^a^ ^32^	38% among >15 years old^a^ ^34^
Status of UNAIDS 90/90/90 targets^d^	83% of the HIV+ aged 16 to 64 years in the study population knew their status; of those 87% were taking ART; of those, 96.5% were virally suppressed (HIV RNA<400 copies/ml). The estimated population‐level viral suppression among HIV+ adults was 70%^a^ ^30^.	Estimates are unavailable at this time	SA: Estimates are unavailable at this time Z: 54% of the HIV+ aged ≥18 years old in the study population knew their HIV+ status; of those 81% were taking ART^a^ ^46^.	68% of the HIV+ in the study population knew their status; of those 80% had received ART; of those, 86% were virally suppressed (HIV RNA<500 copies/ml). The estimated population‐level viral suppression among HIV+ adults was 47% at baseline^a^ ^32^	At the beginning of the trial, 85.8% were estimated to be diagnosed; of those, 37.1% to be engaged in care and actively on ART; on those 77.8% with a documented viral load and virally suppressed 47. Note that trial start corresponds to different time points as all clusters were not opened at same time.

a Denotes the research study population, which is the population in which the primary outcome and main secondary outcomes (among adults) are being measured. (The eligibility criteria for the research study population are specified in Table 3). Data were collected at baseline by the trial team and extracted from publications and conference abstracts.

b Denotes the population living in the trial area; data collected at baseline by the trial team.

c Denotes the populations of the countries in which the trials have been implemented; data from national surveys. IQR: Interquartile range; SA: South Africa; Z: Zambia; K: Kenya; U: Uganda; SW‐U: Southwestern Uganda; E‐U: Eastern Uganda.

d Quantitative comparison of the estimates 90‐90‐90 UNAIDS targets at baseline should be performed cautiously, as there is no standard methodology across trials to compute these figures and is the focus of a separate cross‐trial collaboration.

### 3.2 Trial research designs

As presented in Table 2, the five UTT trials share a common primary aim, which is to assess the impact of UTT on the HIV epidemic. Each trial compares outcomes between communities where all HIV‐infected persons are offered ART regardless of immunological status or clinical stage (among other interventions) and communities where HIV‐infected persons receive ART according to the national guidelines. All trials examine the long‐term benefits and sustainability of the UTT intervention with their extended observation periods (≥3 years). With this common framework, the trials differ somewhat in their designs, with four adopting a community‐randomized design and one adopting a stepped‐wedge clinic‐randomized design. The trials also differ in the number of study arms, with four trials designed with two arms and one trial with three arms.

**Table 2:** Trial research methodologies

Trial	Countries	Design	Primary objective	Primary outcome	Study power	Coefficient of variation between clusters	Estimated trial impact	Duration (average follow‐up)^a^	Estimated completion date
BCPP	Botswana	2 arm community‐level pair‐matched cluster‐randomized trial	To evaluate whether expanded ART initiation (initially by high HIV‐1 RNA level and higher CD4 threshold, now universal ART), combined with strengthened and expanded HIV testing/linkage and male circumcision services, can significantly reduce cumulative HIV incidence at population‐level, in 16 to 64 year old community residents in Botswana.	Cumulative HIV incidence	86% (original parameters and ART initiation criteria) 72% (revised parameters based on observed data, and actual ART initiation criteria over time)	0.26	40% reduction of cumulative HIV incidence in the intervention arm	60 months (average follow‐up of 36 months)	October 2018
*Max*ART	Swaziland	2 arm clinic‐catchment level ‐ stepped‐wedge cluster‐randomized trial	To evaluate whether early ART initiation for all HIV+ individuals can improve retention and viral suppression at population‐level, in over 18‐year‐olds, in Swaziland's government‐managed health system.	Retention and viral suppression	80%	0.5	6% increase in 1‐year retention rate between the control phase and intervention phase	36 months	August 2017
PopART	South Africa, Zambia	3 arm community‐level triplet‐matched cluster‐randomized trial	To determine the impact of two community‐level combination prevention packages, both of which include universal HIV testing and intensified provision of HIV ART and care, on population‐level HIV incidence	Cumulative HIV incidence	>99% for Arm A versus Arm C comparison (original model parameters and ART initiation criteria); In range 44% to 95% for Arm A versus Arm C comparison (revised parameters based on trial data including on testing and treatment coverage over time)	0.15 to 0.2	60% to 65% reduction in HIV incidence in arm A in years 2 and 3 of intervention (original parameters and ART initiation criteria); 35% to 45% reduction of HIV incidence in arm A in years 2 and 3 of intervention (revised parameters)	54 months (average follow‐up of 36 months)	June 2018
SEARCH	Uganda, Kenya	2 arm 2 phase community‐level pair‐matched cluster‐randomized trial	Phase 1: To quantify the impact of early HIV diagnosis and immediate ART using a streamlined care delivery system on the three‐year cumulative HIV incidence among adults aged ≥15 years old in rural communities in Uganda and Kenya Phase 2: To quantify the impact of targeted PrEP, targeted HIV testing and targeted care interventions on top of universal treatment and streamlined care on the 3‐year cumulative HIV incidence among adults aged ≥15 years old in rural communities in Uganda and Kenya	Cumulative HIV incidence	80% in both Phase1 and Phase2	≤0.4	40% reduction in cumulative HIV incidence (both Phase 1 and 2) in the intervention arm	72 months (follow‐up of 36 months in Phase 1 and 36 months in Phase 2)	June 2017 for Phase1 June 2020 for Phase2
ANRS TasP	South Africa	2 arm community‐level cluster‐randomized trial	Phase 1: To estimate the feasibility and acceptability of early ART initiation for all HIV+ individuals combined with six monthly home‐based HIV testing of all adult members of a community and linkage‐to‐care services at population‐level in 16 and above years of age, in KwaZulu‐Natal province in South Africa. Phase 2: To compare how early ART initiation for all HIV+ individuals combined with six monthly home‐based HIV testing of all adult members of a community and linkage‐to‐care services can impact on the reduction in incidence of new HIV infections at population‐level in 16 and above years of age, in KwaZulu‐Natal province in South Africa.	Cumulative HIV incidence	80%	0.25	A 34% reduction in cumulative HIV incidence in the intervention arm	52 months (average follow‐up of 36 months)	June 2014 for Phase 1 July 2016 for Phase 2

ART, antiretroviral therapy; PrEP, pre‐exposure prophylaxis.

a The trial duration refers to the period between the first day an intervention was provided to a study participant and the last day an intervention was provided to a study participant. The average follow‐up refers to the average time a study participant was observed in the context of the trial.

With study‐specific definitions of residency, Table 3 shows that the selection of the trial clusters (i.e. communities or clinic‐catchment areas) were based on diverse criteria. All trials made efforts for their clusters to be large (to ensure that most sexual contacts occur within the cluster) and dispersed (to minimize contamination and overlapping catchment of different health facilities). Two out of the five trials are implementing a pair‐matched design, and one trial is triplet‐matched. With study‐specific definitions of residency, the trial population and eligibility criteria and sizes varied greatly between studies. All trials carefully defined the population that was eligible for the trial interventions (intervention population) which ranged from 4000 to a million of individuals. They collect specific information on selected samples (research study population) and measure the primary outcome through more intensive observations (evaluation population). The study settings are all characterized by some degrees of mobility in and out of the study communities. (Migration rates and characteristics of migrants will be reported in trial‐specific publications.)

**Table 3:** Trial population characteristics at baseline

	BCPP	*Max*ART	PopART	SEARCH	ANRS TasP
Cluster selection criteria	Located in one of three populated geographical regions of Botswana Geographically distinct communities, reachable by road Containing a government primary health care facility (clinic or hospital) which provides ART and PMTCT Community size 2500 to 15,000 (ideal 6000) Not site for other research activities that could impact the trial outcomes	Located in the Hhohho region in Swaziland Catchment area of a government primary health care facility which provides HIV care Not a site for another research activity that could impact the trial outcomes	Located in Western cape province of South Africa or Zambia Non‐adjacent communities Catchment area of a government primary health care facility which provides HIV and TB care Population ≥20,000 High HIV prevalence Not site for other research activities that could impact the trial outcomes	Located in Southwestern, Eastern Uganda or Western Kenya Non‐adjacent communities Served by a government primary health care facility which provides HIV care Accessibility to health centre via maintained transportation route Between 9000 and 11,0000 individuals, not in an urban setting i.e. ≤100,000 Not site for other research activities that could impact the trial outcomes	Located in the Hlabisa sub‐district in KwaZulu‐Natal province, South Africa Neighbouring communities with clear geographical boundaries and distinct social identities ≥15 km^2^ Not site for other research activities that could impact the trial outcomes
Cluster matching and/or stratified randomization and/or restricted randomization criteria	Matching: geographical location, community size, population age structure, baseline access to health services including ART Randomized within matched pairs	Matching: geographical location, clinic‐catchment size Stratified randomization	Matching: geographical location, HIV prevalence Restricted randomization: Community size, ART uptake, HIV prevalence	Matching: geographical location (Phase1 and 2), population density (Phase1), migration (Phase1), occupation (Phase1), number of trading centres (Phase1), Phase 1 intervention arm (Phase2), other drivers of HIV incidence (Phase2) Randomized within matched pairs	No matching Stratified randomization: HIV prevalence
Number of clusters	30	14	21 (12 in Zambia and 9 in South Africa)	32 (10 in Southwestern Uganda, 10 in Eastern Uganda and 12 in Kenya)	22
Average cluster population size [range]^a^	5785 [2748 to 12,865]	8553 [5326 to 14,868]	44,000 [~16,000‐~100,000]	10,450 [8401 – 12,990]	1284 [324 to 2861] (for those aged ≥16 years old)
Intervention population size^b^	105,000 (age 16 to 64 years)	4501	~1000,000	334,512	28,260
Intervention population eligibility criteria^b^	Gave verbal informed consent Is aged ≥16 years Is present at least three nights per month on average in the community over the prior 12 months^a^ Is a Botswana citizen (or spouse of citizen)^e^	Gave verbal informed consent Is aged ≥18 years Is attending one of the study facilities Is not pregnant or breastfeeding at time of enrolment Is HIV‐positive and ART naïve	Gave verbal informed consent Is aged ≥18 years (Year 1, first months of Year 2); all ages (1 October 2015 onwards)	Gave verbal informed consent Is a member of a study household in the trial area	Gave a written informed consent Is aged ≥16 years Is a member of a study household in the trial area
Research study population size^c^	22,000	4501	43,601	146,906	28,260
Research study population eligibility criteria^c^	Gave written informed consent (or parental permission and child assent if <18 years old) Is aged 16 to 64 years Is present at least three nights per month on average in the community over the prior 12 months Is a Botswana citizen (or spouse of citizen) Is present in one of the 20% randomly selected study households Is not currently incarcerated	Gave written informed consent Is a participant to the intervention population	Gave written informed consent Is a member of the trial population Is aged ≥18 and ≤44 years Is present in one of the 2500 study households randomly selected in each cluster; and intending to remain so for the next 3 years Is not currently enrolled in another ART or PreP study Has not been or is not currently enrolled in an HIV vaccine study	Gave verbal informed consent Is a member of the intervention population Is aged ≥15 years at baseline Is present in a study community for ≥6 months of the year prior the study start	Is a member of the intervention population
Evaluation population size^d^	9000	4501	~33,000	118,038	17,659
Evaluation population eligibility criteria^d^	Is a member of the research study population Is HIV‐negative at baseline	Is a member of the research study population	Is a member of the research study population Is HIV‐negative	Is a member of the research study population Is HIV‐negative at baseline	Is a participant to the research study population Is HIV‐negative at baseline

PMTCT, prevention of mother‐to‐child transmission; ART, antiretroviral therapy; PrEP, pre‐exposure prophylaxis.

a Cluster population: Total population of a trial cluster (estimated with baseline data).

b Intervention Population: Population that is eligible for trial interventions, which for purposes of this definition are testing services in the ANRS TasP, BCPP, PopART and SEARCH trials, or HIV treatment services in the *Max*ART trial (estimated with baseline trial data).

c Research study population: Population in which the primary outcome and main secondary outcomes (among adults) are being measured (estimated with baseline data).

d Evaluation population: Population in which the trial impact is being evaluated i.e. in which the primary outcome is being measured (estimated with baseline data).

e In the BCPP trial, being a Botswana citizen (or spouse of a Botswana citizen) and being present at least three nights per month on average in the community over the prior 12 months are eligibility criteria to access early ART but not to access HIV testing and counselling).

### 3.3 Trial intervention packages

As displayed in Table 4, the administration of universal ART is randomized at the community or clinic‐level in all trials. Participants in the intervention arms are offered ART initiation regardless of immunological status or clinical stage. Initially BCPP participants were offered ART if CD4 count ≥350 cells/μl and HIV‐1 RNA ≥10,000 copies/ml, or if CD4 count <350 cells/μl regardless of HIV‐1 RNA. However, with emerging data and evolving WHO recommendations, all HIV‐infected participants are now offered ART. In the control arms of all trials, ART initiation follows national guidelines. In the intervention arms of some trials, additional services are provided, enhanced and/or delivered according to the principles of differentiated care 48, 49, 50, 51: activities to engage the community in accessing HIV care (e.g. roadshows), intensive HIV voluntary testing options (e.g. mobile testing, home‐based testing), intensified health prevention services (e.g. voluntary medical male circumcision (VMMC), screening for HIV‐related diseases and non‐communicable diseases), and support activities for linking and staying in care (e.g. counselling, short message service clinic appointment reminders, follow‐up phone calls). These intervention packages emphasize the key role of an early ART initiation in a wider combination prevention strategy. Furthermore, only a small proportion of those activities is offered in the national standard of care (or offered on a much smaller scale); thus the overall quality and in some cases, efficiency of care delivered to study participants is increased relative to the general population.

**Table 4:** Trial interventions

	BCPP			*Max*ART			PopART				SEARCH			ANRS TasP		
	I	C	S	I	C	S	A	B	C	S	I	C	S	I	C	S
Community mobilization																
Specific mobile activities in the community (e.g. roadshows, public announcements from vehicles, door‐to‐door communication)	x^a^	x	N.A.	**x**	**x**	N.A.	x	x		N.A.	x^a^	x	N.A.	x	x	N.A.
Communication through existing community platforms (e.g. community meetings, civic society activities, schools, clinics)	x^a^	x	N.A.	**x**	**x**	N.A.	x	x	x	N.A.	x^a^	x	N.A.	x	x	N.A.
Health prevention																
Distribution of condoms	x^a^	x	x			x	x	x	x	x	x^a^	x	x	x	x	x
Male circumcision	x^a^	x	x			x	x	x	x	x			x			x
PrEP											x^f^					
PMTCT Option B+	x^a^	x	x			x	x	x	x	x	x^a^	x	x	x	x	x
STI screening	x	x	x			x	x	x		x			x	x	x	x
STI treating	x	x	x			x				x			x			x
TB screening	x^a^	x	x			x	x	x		x	x^a^	x	x	x	x	x
TB treating	x	x	x			x				x	x^a^	x	x			x
Cervical cancer screening			x			x				x	x^a^	x	x	x	x	x
Other HIV opportunistic infections screening	x	x	x			x				x	x^a^	x	x	x	x	x
Screening for chronic illnesses (diabetes and/or hypertension)	x	x	x			x				x	x^a^	x	x	x	x	x
Child care (e.g. immunization, deworming)	x	x	x			x				x	x^a^	x	x			x
Family planning	x	x	x			x				x	x^a^	x	x			x
Antenatal and postnatal care	x	x	x			x				x			x			x
Others (e.g. malaria screening and/or treatment, urgent care, men's health services, dermatological services)	x	x	x			x				x	x^a^	x	x			**x**
HIV testing																
Voluntary HIV testing and counselling	x^a^	x	x	x	x	x	x	x		x	x^a^	x	x	x		x
Provider‐initiated HIV testing and counselling in health facilities	x^a^	x	x			x				x	x^a^	x	x			x
Home‐based HIV testing	x^a^	x^b^					x	x			x^a^	x		x	x	
HIV testing in mobile units	x										x^a^	x		x^f^	x^f^	
HIV testing during mobile community campaigns	x						x	x			x^a^	x				
HIV treatment^c^																
ART initiation of HIV+ individuals with CD4 count ≤500 cells/μl	x	x	x		x	x		x^d^	x^d^	x^d^		x^e^	x		x	x
ART initiation of all HIV+ individuals	x	x	x	x			x	x^d^	x^d^	x^d^	x^a^	x^f^		x		
Linkage to and retention in care																
Clinic referral upon HIV‐positive screen	x	x	x			x	x	x		x	x^a^	x	x	x	x	x
Linkage to care counselling phone calls	x										x^a^	x^f^		x	x	
SMS clinic appointment reminders	x										x^a^	x^f^				
Repeated home visits	x						x	x			x^a^	x^f^		x	x	
Rapid ART initiation (e.g. on the same day as HIV diagnosis)	x			x							x^a^	x^f^				
Adherence support (e.g. support group)	x	x	x				x	x			x^a^	x^f^				
Convenient ART refill process	x										x^a^	x^f^				
Non‐cash incentive (e.g. phone airtime)	x															
Transportation voucher	x	x									x^a^	x^f^				
Point‐of‐care CD4	x	x^b^		x	x						x^a^	x	x	x	x	
Viral load monitoring	x	x	x	x	x					x	x^a^	x^f^	x	x	x	x

**Figure 1:**
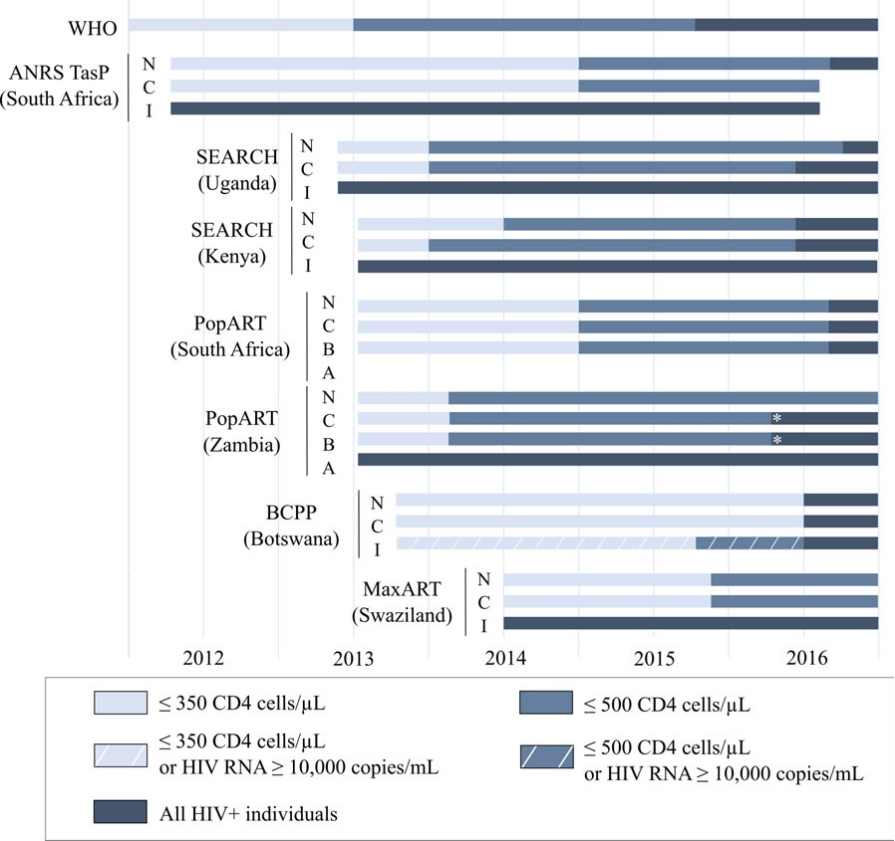
Evolution of the ART eligibility in the UTT trial areas from 1 January 2012 to 31 December 2016. WHO, World Health Organization; N, National recommendations; C, Control arm of the trial; I, intervention arm of the trial; A, Intervention arm A in the PopART trial; B, Intervention arm B in the PopART trial, *shift to universal antiretroviral therapy in the PopART communities in Zambia as part of a pilot for a national roll out.

This table displays information which is valid up to July 2016. S, Services that should be available in both intervention and control arms as the standard of care according to the national guidelines; C, Services delivered in the trial control arm; I: Services delivered in the intervention arm in all trials except PopART; A, Services delivered in the intervention arm A in PopART; B, Services delivered in the intervention arm B in PopART; N.A., Non applicable. PrEP, pre‐exposure prophylaxis; PMTCT, prevention of mother‐to‐child transmission; STI, sexually transmitted infections; SMS, short message service.

a Enhanced provision of these services in the Intervention arm as compared to the Standard of care (S) and Control arms (C).

b In the BCPP trial, the home‐based testing services including point‐of‐care CD4 are available to 20% of the study population in control communities, and 100% in intervention communities.

c The details of treatment eligibility at different times are to be found in Figure 1.

d In the PopART trial, the control arms B and C provide ART initiation of all in Zambia and ART initiation with CD4 count ≤500 cells/μl in South Africa.

e Services delivered during the phase 1 of the SEARCH trial.

f Services delivered during the phase 2 of the SEARCH trial.

Health services are delivered through three main means. First, each trial community is serviced by clinics that provide most of the conventional health activities including HIV care and treatment. In BCPP, *Max*ART, PopART and SEARCH, the trial clinics are government‐led (with strengthening of government staff by some trials), whereas in TasP, the clinics are built for the purpose of the trial to bring services closer to people's homes. Second, health services are also delivered through community‐based activities like home visits (BCPP, PopART, ANRS TasP, SEARCH), HIV testing in mobile units (BCPP, ANRS TasP), and/or health campaigns (SEARCH). Finally, in some instances, trial participants are referred to government‐led health facilities to receive standard of care services that the trials do not provide (e.g. VMMC, sexually transmitted infections care, tuberculosis treatment). The models of care provide examples of how to adapt services to meet the specific needs of patients and the local capacity of the health systems.

### 3.4 Trial themes

Table 5 outlines the main themes that are explored in the trials. The scope of the data collected underlines the trials' potential to explore a range of issues including and beyond HIV incidence. All trials carefully monitor the uptake of trial services and the implementation of the HIV care cascade 52: HIV testing, linkage to care activities, treatment initiation, adherence, and/or viral suppression. Such data will help to assess the trial's success in reaching the UNAIDS 90‐90‐90 targets for 2020. In addition, all trials document individual health outcomes (some through accessing routine programmatic clinical data). At least a subset of trial participants is observed via repeated screening and diagnostic tests, which may occur in the clinic (BCPP, *Max*ART, PopART, ANRS TasP), at home (BCPP, PopART, ANRS TasP) or at community health campaigns and home (SEARCH). Data such as individual CD4 counts, HIV RNA metrics and results of HIV drug resistance tests, are being accumulated on more than a million people across the trials. Some studies are also using HIV viral phylogenetics to investigate transmission patterns at baseline and over time (BCPP, PopART, SEARCH and ANRS TasP). Importantly, the trials also record the costs of the interventions implemented, and will model their cost‐effectiveness, and assess their impact on the operating health systems. The collection of data on human resources, programmatic costs or length of waiting time in clinics will aid in the assessment of the feasibility of integrating universal ART in local health systems. Finally, all trials are investigating social outcomes including facilitators and barriers to accessing HIV care. Besides characterizing the trial populations with standard socio‐demographic information, these trials also collect experiences and perceptions of a sample of trial participants, community members and services providers. Such qualitative research provides evidence to understand the acceptability of the UTT strategies by the general population 29, 53, 54, 55, 56, 57, 58, 59, 60, 61, 62, 63, 64, 65, 66, 67, 68, 69. Overall, the analysis of the various themes addressed within these five trials will contribute to understanding the impact of UTT strategies in various contexts as well as inform the generalization of such a strategy to new contexts.

**Table 5:** Themes explored in the collected trial data

	BCPP	*Max*ART	PopART	SEARCH	ANRS TasP
Implementation of the UTT care cascade					
HIV testing	x		x	x	x
Linkage to care	x		x	x	x
Retention in care	x	x	x	x	x
ART initiation and adherence	x	x	x	x	x
Other HIV prevention services (e.g. PrEP, MC)	x		x	x^a^	
Community's health					
HIV disease progression (according to CD4 cell counts and/or HIV viral loads)	x	x	x	x	x
AIDS or TB or other opportunistic infections (total number of cases and incident cases)	x	x	x	x	x
Other health problems (e.g. diabetes, hypertension chronic kidney disease) (total number of cases and incident cases)^b^	x			x^b^	x
ART‐associated toxicity and adverse events	x	x	x	x	x
Mortality (overall, HIV‐related, child and/or maternal)	x	x	x	x	x
Mother‐to‐child HIV‐1 transmission				x	
Community‐level HIV RNA metrics (e.g. community viral load, proportion of individuals with undetectable viral loads)	x	x	x	x	x
HIV drug resistance (transmitted or acquired virus mutations)	x	x	x	x	x
HIV phylogenetics (e.g. direction of HIV‐transmission events)	x		x	x	x
Economic and health system impact OF UTT					
Cost per patient per year	x	x	x	x	x
Cost‐effectiveness (e.g. modelled cost per infection averted)	x	x	x	x	x
Heath system measures (e.g. time spent from clinic check‐in to completion of clinic visit)		x	x	x	x
Community's experiences					
Participant social and behavioural characteristics (e.g. sexual behaviour and prevention practices, quality of life, social networks, experience of HIV‐related stigma)	x	x	x	x	x
Participant perceptions and attitudes towards the trial interventions (e.g. universal ART, overall care delivery, access to medical services)	x	x		x	x
Community awareness of the trial interventions		x	x	x	x
Providers attitudes towards the trial interventions	x	x	x	x	x

UTT, universal testing and treatment; PrEP, pre‐exposure prophylaxis, MC, medical male circumcision.

a Theme explored during the phase 2 of the SEARCH trial.

b In the SEARCH trial, other health problems are assessed in both HIV‐ and non‐HIV‐infected individuals. In the other trials, they are only assessed in HIV‐infected individuals.

### 3.5 Evolutions/adaptations of study design and interventions

Over the past several years, the HIV policy landscape has been in constant evolution, prompting the on‐going trials to modify their interventions and strategies. In particular, the trials responded to the WHO recommendations related to ART initiation, preventing mother‐to‐child transmission and pre‐exposure prophylaxis (PrEP) and considered the UNAIDS 90‐90‐90 targets for 2020. In all studies, the control arms immediately followed the national guidelines. Figure 1 shows the integration of the 2013 WHO ART recommendations in the arms of each trial, with the threshold for ART initiation changing in the control arms from ≤350 CD4 cells/μl to ≤500 CD4 cells/μl: in 2014 for Zambia (PopART), Uganda and Kenya (SEARCH), and in 2015 for South Africa (ANRS TasP, PopART) and Swaziland (*Max*ART). In 2016, the national guidelines moved to universal ART in Botswana (BCPP), Uganda (SEARCH), Kenya (SEARCH) and South Africa (PopART) and both the intervention and control trial arms switched to universal ART in all of these studies. In the same period, other parameters were altered in the trial protocols, especially to adapt to operational challenges (e.g. simplification of procedures of ART delivery, targeting of the most‐at‐risk population for HIV infection). All PopART communities in Zambia shifted to universal ART as part of a pilot for national roll‐out. SEARCH became a two‐phase trial; in the second phase, all communities were provided with universal treatment with streamlined care, and communities were re‐randomized for targeted PrEP and targeted enhanced testing and care. The BCPP trial started delivering streamlined ART initiation and follow‐up in the intervention arm (with same‐day ART initiation and fewer clinical visits for stable, virologically suppressed patients on ART). Overall, the five trials have proved to be extremely adaptive to the moving sub‐Saharan African political, economic and public health environment, preserving their ability to generate rigorous scientific evidence on UTT effectiveness.

## 4 Discussion

We present a comparative assessment of the five ongoing UTT trials in sub‐Saharan Africa. We found substantial commonalities but also differences between trials in their multidisciplinary activities. In particular, the trials' principal aims and methodologies align in the following strategies, opportunities and goals.

### 4.1 The UTT trials make a plea for combination prevention

The trials deliver a wide range of biomedical, behavioural and structural interventions services to the communities. These strategies build on the plea for combination efforts for HIV prevention and care, which have progressively been gaining influence among scientists and policy makers 63, 70, 71, 72. While the UTT interventions have been designed to both lessen the burden of HIV infection and reduce vulnerability to HIV acquisition, their effect in real‐world situations remains to be proven. The ANRS TasP trial, the first UTT trial to yield final results, did not demonstrate an effect of offering immediate ART on HIV incidence 73, highlighting the key importance of achieving high rates of linkage to care following HIV diagnosis. The SEARCH trial, still ongoing, recently reported having nearly doubled HIV viral suppression within their study population over two years (from 44.7% to 80.2%), surpassing the UNAIDS 90‐90‐90 targets 74. Understanding the magnitude of the effect achieved according to the local context is precisely the aim of the trials. Our appraisal therefore emphasizes the critical monitoring of all the trial services, in order to understand gaps and support the implementation of combination interventions.

### 4.2 The UTT trials optimize the HIV care cascade

All trials also consider the optimization of the whole HIV care cascade as key for a successful UTT strategy. Even though the overall trial attention remains on HIV incidence, key secondary outcomes related to the HIV care cascade components have progressively shown their significance when interpreting the trial impact on the HIV epidemic 3, 75, 76. In their initial designs, all trials underscored that UTT should aim to increase demand for HIV testing, expand HIV testing services, improve linkage to and retention in care as well as adherence to treatment 77, 78. Interestingly, this focus was subsequently formalized as the 2014 UNAIDS 90‐90‐90 targets for 2020. As a consequence, even though the trials were underway before the release of the UNAIDS targets, their results may make an important contribution in demonstrating if and how UTT can help to reach those targets and to what degree those targets might prompt a reduction of new HIV infections, in somewhat different HIV epidemic contexts.

### 4.3 The UTT trials represent an opportunity to design innovative models of HIV care

In the HIV/AIDS field, models of care have constantly evolved and adapted to the changing needs of communities, patients and to new scientific evidence 79. Recently, a flurry of new, more‐efficient HIV care models has been proposed which challenges the classic standard facility‐based model. Among them, the scale‐up of ART is leading to one of the most sweeping changes in health care delivery, especially in sub‐Saharan Africa 10, 80, 81. In this comparative analysis, we document the pragmatic systems and processes of UTT care models that are being field‐tested in sub‐Saharan Africa and are thus on the forefront of large‐scale practice. The trials showcase key interventions which could be integrated in the new models of care, namely community‐based approaches for HIV testing with the use of point‐of‐care HIV tests, rapid facility‐based provision of ART, community ART delivery, active follow‐up activities, integrated chronic care model with HIV and non‐communicable diseases, effective community engagement and close collaboration with the existing health services.

### 4.4 The UTT trials give insights on how to generalize UTT to various contexts

To organize health services, including scaled‐up access to HIV care and treatment, the trials embrace HIV care approaches that are differentiated by patient type, are people‐centred and integrated across diseases and health systems functions. As the recognition of differentiated care and integrated care is growing 48, 49, 50, 51, all studies have embraced a common principle, that contextualization is imperative, with integrated services being beneficial in some settings and differentiated care being required in other settings. All trials have chosen their UTT models of care, based on the contexts in which they were implemented, including the possibility of being integrated in the local health care delivery system. Understanding and accounting for the heterogeneous social, political, economic, historical and health systems contextual factors that shape whether and how these UTT strategies work is deemed essential prior to a full‐scale rollout of a UTT strategy 59, 82. It is equally important to pay attention to the operational challenges that could result from implementing UTT, including effects of the interventions on individual's everyday lives, health systems and governmental structures 57.

### 4.5 The UTT trials are critical to ensure a dynamic interface between policy and research

During their conduct, all trials have regularly exchanged information with their local and/or national health authorities, especially to communicate the trial progresses and the variety of sources of uncertainties that could influence their outputs. This review is an opportunity to provide policy makers with a comprehensive overview of the characteristics of all trials, which are likely to affect the results of the implemented UTT strategies in different contexts. All trials have efficiently adapted their design and interventions to changing guidelines both for ethical reasons and to ensure that their results are of maximal relevance to the public health landscape. Collectively, the trials represent the most relevant information to help answer policy and resource allocation questions. While the plea for UTT is now clear, many countries still need to evaluate the feasibility and cost of adopting such a strategy into their national health system 13. A clear understanding of the available evidence will be a key for informed policy formulation.

## 5 Limitations

This analysis has several limitations. First, differences in the trials' design and data collection schemes (Table 3) prevented direct comparison of several indicators at baseline (Table 1). To improve comparison, we provided statistics from both data collected at baseline as well as national indicators (Table S1).

Second, the methods applied are not based on a consensual methodology which would allow a standardized and thus validated direct comparison of the trials. Because no such methods exist yet, we developed, as a consortium, a simple way to comparatively assess all trials. The expertise and experience of the researchers was central to decide which trial components should be compared, at what level of detail, and how to interpret their differences across trials. We opted for a comprehensive description of the trials. We acknowledge that more information on the modelling, economic or social perspectives of the UTT strategies is needed to better understand the trials' interim and final results. Such analyses will be the focus of future collaborative work within our consortium.

Third, when faced with this diversity of UTT approaches, our analysis does not answer the questions arising: are certain study designs more relevant than others? Are specific trial interventions more effective than others? With quantitative data not yet available across trials in similar situations, it is not possible to answer these questions. With this caveat in mind, we conclude that the most appropriate UTT interventions will vary by context and suspect that no single best strategy will emerge.

Finally, in this study, we focused on the five controlled trials measuring the impact of service delivery interventions for UTT in sub‐Saharan Africa. We also acknowledge that non‐randomized interventions and other operational research experiences will likely be an important source of information, particularly with regard to lessons learnt for implementation. Other novel service delivery methods for UTT are indeed piloted in observational settings but were not captured in this paper 4. Inclusion of non‐randomized studies and evidence may be a focus of future collaborative work.

## 6 Conclusion

To our knowledge, this is the first detailed exploration of the trials evaluating UTT strategies in sub‐Saharan Africa. This *a priori* exercise, i.e. before the individual study findings are released, is not usually performed by investigators. By providing detailed qualitative and quantitative insights about the different trial approaches, our analysis was intended to prospectively build the foundation for more collaborative research on UTT. It also aimed at providing valuable information as the trials are underway and starting to publish process data and findings, to guide as rapidly as possible the strengthening of services for testing and treatment in sub‐Saharan Africa, for both individual and public health benefits.

The present study reports on a wide range of attributes of the service delivery models for UTT and highlights the diversity in implementation. The population‐level impact of UTT is likely to be context‐dependent. The UT^3^C consortium, with this first paper, intends to set the path for a quicker and deeper understanding on this issue and prepare for future comparison of trial findings and to inform as efficiently as possible public policy in country and internationally.

## Competing interests

The authors declared that they have no competing interests

## Authors' contributions

François Dabis, Joanna Orne‐Gliemann and Delphine Perriat conceived and designed the study. Laura Balzer, Shahin Lockman, Delphine Perriat, Kalpana Sabapathy and Fiona Walsh organized and conducted the data collection. Delphine Perriat wrote the first draft of the manuscript and also produced the figures and the tables. All authors analysed the data, contributed to the writing (and editing) of the manuscript, read and met the criteria for authorship and agreed with manuscript results and conclusions.

## Funding

UT^3^C received technical support from SSS through a grant of the Bill and Melinda Gates Foundation. **The BCPP trial** is supported by the United States President's Emergency Plan for AIDS Relief (PEPFAR) through the Centers for Disease Control and Prevention (CDC) under the terms of cooperative agreement U01 GH000447. **The** ***Max*** **ART trial** is supported by the Dutch Postcode Lottery, the Embassy of the Kingdom of the Netherlands in Mozambique, British Colombia Centre of Excellence in Canada, Mylan, and Médecins Sans Frontières. **The PopART trial** is sponsored by the Division of AIDS, National Institute of Allergy and Infectious Diseases (NIAID), United States National Institutes of Health (NIH), and is funded by the NIAID, the National Institute of Mental Health, the Office of the United States Global AIDS Coordinator, the Bill and Melinda Gates Foundation and the NIH. **The SEARCH trial** sponsors, partners and advisors include the NIAID of NIH, PEPFAR, the Ministries of Health of Uganda and Kenya, the Bill and Melinda Gates Foundation, the WHO, UNAIDS, the World Bank, the Global Fund for HIV, TB and Malaria, the Infectious Diseases Research Collaboration (IDRC), the Kenya Medical Research Institute (KEMRI), and Gilead Sciences, Inc. The research reported in the publication was funded by Division of AIDS, NIAID of NIH under award number U01AI099959 and in part by the PEPFAR and Gilead Sciences. **The ANRS TasP trial** was sponsored by the French National Institute of Health and Medical Research‐French National Agency for AIDS and Viral Hepatitis Research (Inserm−ANRS), and was funded by the ANRS, the Deutsche Gesellschaft für Internationale Zusammenarbeit (GIZ) GmbH, the International Initiative for Impact Evaluation (3iE), Gilead Sciences, Inc and MERCK & Co., Inc.

Research reported in this publication was supported by the trial partners mentioned above. The findings and conclusions in this report are those of the authors and do not necessarily represent the official position of the trial partners. The funders had no role in study design, data collection and analysis, decision to publish, or preparation of the manuscript. The trials gratefully acknowledge the Ministries of Health of Botswana, Kenya, South Africa, Swaziland, Uganda and Zambia, the trial research teams, collaborators and advisory boards, funding agencies, and especially all communities and participants involved.

## Supporting information


**Table S1.** National indicators of the countries of trial implementation
