# Supplementary material for: Comparative assessment of five trials of universal HIV testing and treatment in sub‐Saharan Africa
Source: J Int AIDS Soc. 2018 Jan 8;21(1):e25048. doi: 10.1002/jia2.25048 (PMC5810333; doi:10.1002/jia2.25048)
Supplement: Supplementary file 1 — Table S1. National indicators of the countries of trial implementation [file JIA2-21-e25048-s001.docx]

**Supplementary Table 1: National indicators of the countries of trial implementation**

| National Indicators | Botswana | Swaziland | South Africa | Zambia | Uganda | Kenya |
| --- | --- | --- | --- | --- | --- | --- |
| CENSUS AND DEMOGRAPHIC | | | | | | |
| Population census | 2,021,000 (2013) [[36](#_ENREF_36)] | 1,250,000 (2013) [[37](#_ENREF_37)] | 52,776,000 (2013) [[38](#_ENREF_38)] | 14,539,000 (2013) [[83](#_ENREF_83)] | 34,634,650 (2014) [[84](#_ENREF_84)] | 44,354,000 (2013) [[85](#_ENREF_85)] |
| Rural population (%) | 43 (2013) [[36](#_ENREF_36)] | 79 (2013) [[37](#_ENREF_37)] | 36 (2013) [[38](#_ENREF_38)] | 60 (2013) [[83](#_ENREF_83)] | 81,6 (2014) [[84](#_ENREF_84)] | 75 (2013) [[85](#_ENREF_85)] |
| Population aged [0-14] (%) | 34 (2013) [[36](#_ENREF_36)] | 38 (2013) [[37](#_ENREF_37)] | 30 (2013) [[38](#_ENREF_38)] | 50 (2014) [[86](#_ENREF_86)] | 48 (2014) [[84](#_ENREF_84)] | 43 (2014) [[87](#_ENREF_87)] |
| Population aged [15-34] (%) | 71 (2013) [[88](#_ENREF_88)] | 34 (2007) [[89](#_ENREF_89)] | 33 (2003) [[90](#_ENREF_90)] | 29 (2014) [[86](#_ENREF_86)] | 17,7 (2014) [[84](#_ENREF_84)] | 32 (2014) [[87](#_ENREF_87)] |
| LABOR AND POVERTY | | | | | | |
| Unemployment rate among the working-age population/labor force (%) | 20 [15-64] (2011) [[91](#_ENREF_91)] | 47 (15+) (2013) [[39](#_ENREF_39)] | 25 [15-64] (2014) [[92](#_ENREF_92)] | 40 [15-49] (2014) [[86](#_ENREF_86)] | 58 [14-64] (2014) [[84](#_ENREF_84)] | 67 [15-49] (2014) [[87](#_ENREF_87)] |
| Poverty headcount ratio at national poverty lines %) | 15 (2010) [[40](#_ENREF_40)] | 63 (2009) [[41](#_ENREF_41)] | 54 (2010) [[42](#_ENREF_42)] | 61 (2010) [[43](#_ENREF_43)] | 19 (2012) [[44](#_ENREF_44)] | 46 (2005) [[45](#_ENREF_45)] |
| EDUCATION AND HEALTH | | | | | | |
| Illiteracy rate among adult population (%) | 14 [15-49] (2011)[[91](#_ENREF_91)] | 12 (15+) (2011) [[93](#_ENREF_93)] | 19 [15-49] (2011) [[94](#_ENREF_94)] | 26 [15-49] (2014) [[86](#_ENREF_86)] | 28 (10+) (2014) [[84](#_ENREF_84)] | 11[15-49] (2014) [[87](#_ENREF_87)] |
| HIV prevalence among adults [15-49] (%) | 22 (2015) [[95](#_ENREF_95)] | 29 (2015)[[31](#_ENREF_31)] | 19 (2015) [[96](#_ENREF_96)] | 13 (2015)[[97](#_ENREF_97)] | 7.1 (2015)[[98](#_ENREF_98)] | 5.9 (2015)[[99](#_ENREF_99)] |
| Number of people receiving ART | 223,974 (2013) [[100](#_ENREF_100)] | 100,138 (2013) [[33](#_ENREF_33)] | 1,600,000 (2012) [[101](#_ENREF_101)] | 556,002 (2014) [[102](#_ENREF_102)] | 750,896 (2014) [[103](#_ENREF_103)] | 656,000 (2013) [[104](#_ENREF_104)] |
